# Innocent Heart Murmur

**DOI:** 10.7759/cureus.3689

**Published:** 2018-12-05

**Authors:** Arpan R Doshi

**Affiliations:** 1 Pediatric Cardiology, Children's Mercy Hospitals and Clinics, Wichita, USA

**Keywords:** murmur, innocent heart murmur, functional heart murmur, pediatric murmur, flow murmur, heart murmur

## Abstract

Heart murmur is the most common reason for a referral to a pediatric cardiologist. Virtually all children have a heart murmur during their childhood. Less than 1% of murmurs are pathological in children. Innocent/functional heart murmur is the most common type of heart murmur. There are multiple theories proposed to identify etiology of innocent heart murmur with varying consensus, but everybody agrees that innocent heart murmur does not carry any morbidity or mortality risk. Even today, heart murmur is associated with high physician uncertainty and parental anxiety. Extensive cardiac evaluation for such a benign finding is also associated with high health care utilization and cost. This article attempts to review this long-known finding which continues to remain a diagnostic challenge.

## Introduction and background

Cardiac murmurs have remained a “hot topic” in cardiac evaluation since the invention of the stethoscope by Rene Laennec in the early 17th century [1]. Heart murmur continues to remain the most common reason for referral to pediatric cardiologist [2]. Less than 1% of pediatric heart murmurs are associated with congenital heart disease, and most of the heart murmurs are innocent in nature [3]. Terminologies like “innocent”, “physiologic”, “benign”, “normal” or “functional” are frequently used but calling it “innocent” clearly conveys the non-pathological nature of this finding to the parents and patients.

Murmur is a sound produced by vibrations caused by the flow of blood through the heart [4]. Several different mechanisms are proposed regarding the development of any murmur such as turbulence from the blood flow, cardiac vibrations, Bernoulli effect, eddy current, etc. Dr. George Frederic Still, in the year 1909, first described “musical murmur” in pediatric patients and deemed it as innocent [5].

## Review

Types of innocent heart murmurs

Innocent heart murmurs in children can be categorized under four distinct groups: Still’s vibratory murmur, pulmonary flow murmur, supraclavicular systemic flow murmur, and venous hums. Many high output states like anemia, fever, arteriovenous malformation, etc. can also result in heart murmur [5].

Still’s murmur: McKusick et al. very appropriately suggested the musical instrument, Aeolian Island harp, as an analogue for Still’s murmur [6]. Still’s murmur is a brief, vibratory quality, grade 1-3, midsystolic, and low-pitched murmur. This is best heard with the bell of a stethoscope. This murmur is heard at the left lower sternal border and occasionally radiates to the cardiac apex. Typical age group for this murmur is three years to early adolescence, but it can be present at any age. Because the innocent murmurs are produced by normal flow dynamics, any alteration in the flow with a change in patient position will change the murmur characteristics. This murmur is the loudest in the supine position and it diminishes in intensity when a child is made to sit or stand up, as these positions diminish the venous return to the heart. This could be a valuable tool while examining Still’s murmur in the clinic.

Some of the proposed mechanisms for the development of Still’s murmur are the presence of “fibrous bands” or “false tendon” in the left ventricle [7-8], smaller aortic size causing increase in the flow velocity [9], insertion of tricuspid valve chordae into the right ventricular outflow tract [5], increased left ventricular output with relative bradycardia [10], vibrations from cardiac structures [10], and lower aortic arterial elastance with a higher left ventricular contractility [11]. There is no clear consensus on the mechanism of its origin at this time.

Pulmonary flow murmur: This is a harsh quality, grade 2-3, medium-high pitched, ejection systolic murmur. This murmur is best heard with the diaphragm of a stethoscope. It is thought to originate from the right ventricular outflow. It is best heard at the left upper sternal border over the “pulmonary area” and it often radiates to the back and/or axillae. This murmur can be very prominent in high output states and in patients with pectus excavatum deformity of the chest. Pulmonary flow murmur can be differentiated from pulmonary valve stenosis murmur by its quality and absence of pulmonary valve click. Pulmonary flow murmur is also very responsive to change in flow dynamics. The intensity increases with inspiration and lying down due to an increase in venous return, and decreases with standing up and Valsalva maneuver.

Supraclavicular systemic flow murmur: This is a harsh quality, crescendo-decrescendo, medium-high pitched, grade 2-3 murmur heard over the supraclavicular region with radiation to the carotids. This murmur is best heard with the diaphragm of a stethoscope and can be present in any pediatric age group as well as young adults. In contrast to aortic valve stenosis murmur, this is not accompanied by the systolic click of the aortic valve. This murmur is thought to arise from the normal flow of blood from the aorta into head-and-neck vessels [12]. This murmur is loudest in a supine position and it diminishes in intensity with hyperextension of the neck.

Venous hum: This is a continuous low-pitched murmur best heard over the lower neck, just lateral to the sternocleidomastoid muscles. This originates from the systemic venous return of the superior vena cava. Due to its low-pitch, it is best heard with the bell of a stethoscope. This murmur is very sensitive to position and disappears with making the child look down or to the side. It is most prominent in the sitting position and diminishes or disappears on lying down. This can be easily differentiated by patent ductus arteriosus murmur which is harsh machinery in character and does not change or disappear with changing neck position.

Assess beyond the murmur

A pediatric cardiovascular examination should not primarily focus and end with listening for the presence or absence of heart murmur. A detailed birth history, past medical history, growth chart assessment, detailed family history, and head-to-toe assessment are essential components that help with making a competent clinical diagnosis [3]. Many of the complex and serious cardiovascular abnormalities may not present with a heart murmur. A cardiovascular exam should include inspection and palpitation of precordium, assessment of peripheral pulses, assessment of perfusion, abdominal organomegaly, etc. in addition to auditory assessment. Lastly, auscultation should include assessment of heart sounds (S1 and S2) first before focusing on the heart murmur. In the pediatric population, the sequence of assessment may need to be modified based on the patient’s age and level of cooperativeness.

Impact beyond the patient

Referral of a child to a cardiologist for evaluation of innocent heart murmur has a significant impact that extends beyond the patient. Diagnosis of heart murmur, even if innocent, is associated with significant healthcare resource utilization and parental/caregiver anxiety [13-17]. Appropriate use criteria for initial transthoracic echocardiography were released by the American Academy of Pediatrics, American Heart Association, American Society of Echocardiography, Heart Rhythm Society, etc. in the year 2014 which clearly notes transthoracic echocardiogram for presumed innocent heart murmur as “rarely appropriate” indication [18]. Despite published guidelines, there is a significant utilization of transthoracic echocardiography as well as recurrent cardiology visits for innocent heart murmur [13-14]. Giuffre et al. and Bardsen et al. looked at parental anxiety after a diagnosis of childhood heart murmur [15,17]. Both studies reported significantly higher anxiety level in parents after the initial diagnosis. Even when the parents were given a handout regarding innocent heart murmur prior to the evaluation by a cardiologist, it did not decrease the anxiety significantly. Parental anxiety was significantly relieved after evaluation and counselling by a pediatric cardiologist. It should be further explained to parents that child may never “outgrow” the murmur and murmur may only be intermittently audible.

Role of auditory training and computer-aided auscultation devices

Auscultation skill is the cornerstone of competent cardiovascular examination. Evaluation of heart sounds and heart murmur plays an important role in making an accurate cardiac diagnosis and dictate the management plan. But with modernization and availability of various “gadgets” in medicine, unfortunately, this is becoming a neglected skill [19]. Suboptimal performance by practicing physicians and medical trainees has been well documented in various studies [20-21]. Finley et al. showed in their study that well planned auditory training program rapidly teaches students to distinguish between innocent and pathological murmur with at least 90% accuracy [22]. They also further show that the skill level does decline over time but can be remastered with repeat training. Repetition and practice are the key factors to master auscultation skills [23].

There are a few commercially available softwares in the market designed to aid with determining pathological nature of a heart murmur [24]. These softwares have improved significantly over time but they still lag high specificity and sensitivity for making an accurate diagnosis consistently. Additionally, the patient’s age and heart rate significantly affect its sensitivity and specificity. There softwares put forward a very promising premise for use by frontline providers to differentiate innocent vs pathological murmur with high certainty. But for the time being they are not ready for routine use in author’s opinion.

## Conclusions

In conclusion, innocent heart murmur is a benign/functional sound produced by a flow of blood through the heart. It remains a significant source of diagnostic uncertainty among primary care physicians and it is still the most common reason for a referral to a pediatric cardiologist. Despite being an “innocent finding”, it still remains a significant source of health care resource utilization and parental anxiety.
